# Effects of Endocrine Disruptors *o*,*p′*-Dichlorodiphenyltrichloroethane, *p*,*p′*-Dichlorodiphenyltrichloroethane, and Endosulfan on the Expression of Estradiol-, Progesterone-, and Testosterone-Responsive MicroRNAs and Their Target Genes in MCF-7 Cells

**DOI:** 10.3390/toxics10010025

**Published:** 2022-01-07

**Authors:** Tatiana Kalinina, Vladislav Kononchuk, Lyubov Klyushova, Lyudmila Gulyaeva

**Affiliations:** 1 Federal Research Center of Fundamental and Translational Medicine, Timakova Str. 2/12, 630117 Novosibirsk, Russia; kononchuk@niimbb.ru (V.K.); klyushovals@mail.ru (L.K.); gulyaeva@niimbb.ru (L.G.); 2 Meshalkin National Medical Research Center, Ministry of Health of the Russian Federation, Rechkunovskaya Str. 15, 630055 Novosibirsk, Russia; 3 Institute for Medicine and Psychology, Novosibirsk State University, Pirogova Str. 2, 630090 Novosibirsk, Russia

**Keywords:** organochlorine pesticide, dichlorodiphenyltrichloroethane, endosulfan, hormone receptor, endocrine disruptor, microRNA

## Abstract

Many studies have shown that dichlorodiphenyltrichloroethane (DDT) exposure raises breast cancer risk. Another insecticide with similar properties is endosulfan, which has been actively used in agriculture after DDT prohibition. Previously, we have identified some estradiol-, progesterone-, and testosterone-sensitive microRNAs (miRNAs, miRs). Because DDT and endosulfan have estrogenic, antiandrogenic, and antiprogesterone properties, we hypothesized that these miRNAs are affected by the insecticides. We quantified relative levels of miRNAs and expression levels of their target genes in breast cancer MCF-7 cells treated with *p*,*p′*-DDT, *o*,*p′*-DDT, or endosulfan. We also quantified miR-19b expression, which, as previously shown, is regulated by estrogen. Here, we observed that miR-19b expression increased in response not only to estradiol but also to testosterone and progesterone. Treatment of MCF-7 cells with *p*,*p′*-DDT or endosulfan decreased the protein levels of apoptosis regulators TP53INP1 and APAF1. In cells treated with *o*,*p′*-DDT, the TP53INP1 amount decreased after 24 h of incubation, but increased after 48 h of incubation with insecticide. OXTR expression, which is known to be associated with breast carcinogenesis, significantly diminished under the exposure of all insecticides. In cells treated with *p*,*p′*-DDT or *o*,*p′*-DDT, the observed changes were accompanied by alterations of the levels of hormone-responsive miRNAs: miR-324, miR-190a, miR-190b, miR-27a, miR-193b, and miR-19b.

## 1. Introduction

Over the past 50 years, it has been established that a variety of chemicals can affect the endocrine system [1]. Such compounds are called endocrine-disrupting chemicals (EDCs). They can either mimic endogenous hormones in the body or competitively bind to a hormone receptor [2]. EDCs are usually lipophilic compounds that bioaccumulate in adipose tissue [3]. One of the first EDCs to be studied was dichlorodiphenyltrichloroethane (DDT). It is an organochlorine pesticide that was widely used in the 1940s–1970s in agriculture. At present, DDT is approved for malaria control [4]. Another organochlorine pesticide is endosulfan, which was extensively used in the 1980s and 1990s. Although endosulfan was banned globally in 2011, it is still employed illegally in certain countries [5,6]. Despite the global ban on these pesticides in agriculture, owing to their extensive use in the past and continued use in some countries, these compounds and their metabolites are still detectable in human breast milk and serum [7,8,9].

Isomers and metabolites of DDT and endosulfan may have an estrogen agonist activity and androgen and progesterone antagonist activities [10,11,12,13,14,15]. Several reports point to a link between exposure to these pesticides and a higher risk of malignant tumors [8,16,17,18]. Nonetheless, the mechanisms by which DDT and endosulfan can lead to cancer initiation are not fully investigated. The most studied is the relation of pesticides with the risk of breast cancer, whose initiation and progression are known to be associated with changes in the expression and activity of estrogen receptor (ER), progesterone receptor (PR), and androgen receptor (AR) [19,20,21,22]. The fact that DDT and endosulfan alter the microRNA (miRNA, miR) expression profile also implies that these pesticides can contribute to cancer initiation [23,24,25]. Through effects on the levels of miRNAs, insecticides may change protein expression of many tumor suppressor genes or oncogenes. We have previously identified a number of estradiol-, testosterone-, and progesterone-sensitive miRNAs [26]. Therefore, to search for new targets of pesticides in breast cells, we analyzed the expression of these miRNAs and of their predicted target genes in ER-, PR-, and AR-expressing breast cancer MCF-7 cells treated with various doses of *p*,*p′*-DDT, *o*,*p′*-DDT, or endosulfan. To confirm the role of ER, PR, and AR in the identified changes, we also quantified miRNAs in MDA-MB-231 cells (an ER-, PR-, AR-negative cell line) exposed to *p*,*p′*-DDT, *o*,*p′*-DDT, or endosulfan.

## 2. Materials and Methods

### 2.1. Chemicals and Reagents

4,4′-DDT (*p*,*p′*-DDT; 98%, SKU 386340), 2,4′-DDT (*o*,*p′*-DDT; analytical standard, SKU 49018), endosulfan (analytical standard, SKU 32015), estradiol, progesterone (≥99%, SKU P0130), and testosterone (≥99%, SKU 86500) were purchased from Sigma-Aldrich (St. Louis, MO, USA).

### 2.2. Cell Culture

The ER-, PR-, AR-positive breast cancer MCF-7 cell line and ER-, PR-, AR-negative breast cancer MDA-MB-231 cell line were obtained from the Russian Cell Culture Collection (a St. Petersburg (Russia) branch of the European Tissue Culture Society). MCF-7 cells were cultivated in Iscove’s modified Dulbecco’s medium (IMDM; Gibco BRL Co., Gaithersburg, MD, USA) supplemented with 10% of fetal bovine serum (Gibco BRL Co.), 2 mM l-alanyl-l-glutamine (Gibco BRL Co.), 250 mg/mL amphotericin B, and 100 U/mL penicillin/streptomycin (Gibco BRL Co.). MDA-MB-231 cells were grown in Dulbecco’s Modified Eagle Medium/Nutrient Mixture F-12 media (DMEM/F-12; Gibco BRL Co.) with 10% FBS (Gibco BRL Co.), 2 mM l-alanyl-l-glutamine (Gibco BRL Co.), 250 mg/mL amphotericin B, and 100 U/mL penicillin/streptomycin (Gibco BRL Co.). The cells were maintained in a 5% CO_2_ incubator at 37 °C and passaged when 65–80% confluent. Conventional PCR assays were performed to verify the absence of mycoplasma contamination. The treatment of cells with estradiol, progesterone, testosterone, *p*,*p′*-DDT, *o*,*p′*-DDT, or endosulfan was carried out in accordance with a protocol published elsewhere [27].

### 2.3. Viability, Apoptosis and Proliferation Assay

Cell viability, apoptosis and proliferation were detected by Hoechst 33342/propidium iodide (PI) staining as previously described [28]. The cells were treated for 6, 24 and 48 h with insecticides dissolved in DMSO at concentrations 0.1–50 μM. Treated cells and control cells were stained with Hoechst 33342 (Sigma-Aldrich) for 30 min at 37 °C and PI (Sigma-Aldrich) for 10 min at 37 °C. An IN Cell Analyzer 2200 (GE Healthcare, Chicago, IL, USA) was used to perform automatic imaging of four fields per well under 200× magnification, in brightfield and fluorescence channels. The images produced were used to analyze live, apoptotic and dead cells among the whole population using the IN Cell Investigator software (version 1.5, GE Healthcare, Chicago, IL, USA. All data shown are means of three wells. Quantitative data are expressed as the mean ± standard deviation (SD). The cytotoxic activity was determined as half-maximal lethal concentration (LC_50_), which was defined as ‘the compound concentration that reduces the number of live cells by 50%’ and was calculated from curves constructed by plotting cell survival (%) versus drug concentration (µM). The proliferation activity was determined as half-maximal inhibitory concentration (IC_50_), which was calculated from a curve constructed by plotting a cell count (%) versus drug concentration (µM).

### 2.4. RNA Isolation

Total RNA was isolated using the TRIzol™ Reagent (Invitrogen, Carlsbad, CA, USA). Glycogen (Amresco, Cleveland, OH, USA) served as an RNA co-precipitant. RNA integrity was evaluated by agarose gel electrophoresis. Concentration and purity of RNA were assessed on an Agilent-8453 spectrophotometer (Agilent Technologies, Santa Clara, CA, USA) at wavelengths 260 and 280 nm.

### 2.5. Reverse Transcription and Real-Time PCR (RT-PCR) of MiRNAs

Stem-loop primers [29] and the RT-M-MuLV-RH Kit (Biolabmix Ltd., Novosibirsk, Russia) were used for reverse transcription of miRNAs. PCR was conducted with TaqMan probes and the BioMaster UDG HS-qPCR (2×) Kit (Biolabmix Ltd.). Detection of PCR products was carried out by means of a CFX96™ Detection System (Bio-Rad Laboratories, Hercules, CA, USA). Expression levels of small nuclear RNAs U44 and U48 were used to normalize the data.

The primer sequences for the reverse transcription are listed in Table 1.

The oligonucleotides that were employed for PCR are listed in Table 2.

The reverse primer targeting the stem-loop region in the synthesized cDNAs was 5′-AGTGCAGGGTCCGAGGTA-3′ (except for the cDNA of U48). Each sample was analyzed in triplicate (technical replicates). The fold-change of each miRNA was calculated using the threshold cycle (CT) method (2^−∆∆Ct^).

### 2.6. MRNA Reverse Transcription and RT-PCR

Reverse transcription of mRNA was performed by means of the RT-M-MuLV-RH Kit (Biolabmix Ltd.), and 0.8 μg of RNA was used in this reaction. PCR was carried out with the BioMaster HS-qPCR SYBR Blue(2×) (Biolabmix Ltd.) reaction mix, followed by loading into the CFX96™ Detection System (Bio-Rad Laboratories). *GAPDH*, *SYMPK*, and *ANKRD17* served as reference genes. The specific primers that were used in PCR are listed in Table 3.

The optimal concentration of each primer was 250 nM. Each PCR was carried out with 0.3 μL of cDNA in a final reaction volume of 20 μL. Melting profiles were employed to assess PCR specificity. Samples were analyzed in triplicate (technical replicates). The relative gene expression was analyzed using the 2^−∆∆Ct^ method.

### 2.7. Western Blot Analysis

The cells were washed with PBS and lysed in mRIPA buffer according to a previously published protocol [30]. The protein concentration was determined using the Pierce™ BCA Protein Assay Kit (Thermo Fisher Scientific, Waltham, MA, USA; cat. # 23225). Protein samples (20 μg per lane) were separated by SDS-PAGE and transferred to a polyvinylidene difluoride membrane. The membranes were blocked in PBS containing 5% of nonfat milk and 0.1% of Tween 20 and probed with primary antibodies. The following specific antibodies were utilized in this work: anti-p53 DINP1 (cat. # ab202026, Abcam, Waltham, MA, USA), anti-APAF1 (ab2001), anti-Oxytocin Receptor (ab217212), anti-PTPRS (ab55640), anti-GAPDH (ab181602), a goat anti-mouse IgG H&L (HRP) antibody (ab97040; secondary antibody), and a goat anti-rabbit IgG H&L (HRP) antibody (ab97051; secondary antibody). The bands were visualized using the Pierce™ ECL Western Blotting Substrate (Thermo Fisher Scientific). The intensity values of the bands were quantified in the GelQuant.NET software provided by biochemlabsolutions.com (version 1.8.2) [31].

### 2.8. Bioinformatic Analysis

To determine whether the promoter region of the *MIR19b* gene contains a sequence matching the binding site of AR and PR, a putative miRNA gene promoter region was retrieved from the human genome (hg38) 10,000 nucleotides upstream from the start of a precursor miRNA sequence according to MirGeneDB [32]. AR- and PR-binding sites were found in these regions using a position weight matrix (MA0007.2, MA0113.3) from Jaspar (http://jaspar.genereg.net/, accessed on 12 December 2017) [33] (sequences of binding sites for these receptors are identical) in Biostrings (R Bioconductor package) [34].

### 2.9. Statistical Analysis

STATISTICA software (version 12; TIBCO Software Inc., Palo Alto, CA, USA) was used for statistical data analysis. The data are displayed as means. Statistical comparisons between groups were performed by one-way analysis of variance (ANOVA), followed by Dunnett’s multiple comparison post hoc test. Significance was set to *p* < 0.05.

## 3. Results

### 3.1. The Effects of p,p′-DDT, o,p′-DDT, and Endosulfan on Viability and Proliferation of MCF-7 Cells

The effects of *p,p′*-DDT, *o,p′*-DDT and endosulfan on viability of MCF-7 cells was evaluated by dual staining with Hoechst 33342/propidium iodide (PI). The cytotoxic activity was determined as LC_50_, and the proliferation activity as IC_50_. The LC_50_ values after incubation of the cell lines with the tested compounds for 6, 24 and 48 h are listed in Table 4, and the IC_50_ values are presented in Table 5.

The choice of DDT doses (0.1 μM and 10 μM) for further investigation of miRNA expression was based on the results of previous studies [24,35]. Since endosulfan, in comparison with DDT, had a high cytotoxic activity and a significant effect on cell proliferation, we incubated cells with 0.1 and 1 μM of this insecticide. We have previously demonstrated that after 6, 24, and 48 h of incubation with DDT or endosulfan, there is a change in the expression of target genes of ER, PR, and AR [27]. Therefore, to study the effect of insecticides on the expression of hormone-responsive miRNAs, we chose the same incubation time.

### 3.2. ER-, PR-, and AR-Dependent Changes in the Expression of Hormone-Responsive MiRNAs in Cells Treated with DDT or Endosulfan

In an earlier study, we identified many miRNAs whose promoter regions contain ER-, PR-, and/or AR-binding sites and showed that the expression of some of them in MCF-7 cells is sensitive to estradiol, progesterone, or testosterone [26]. Table 6 presents a list of these miRNAs and compounds that alter their expression. Here, we also investigated the effect of hormones and insecticides on the expression of miR-19b. It was previously shown that this miRNA is estrogen-regulated [36]. Using bioinformatic analysis, we also found that the hsa-mir-19b-1 promoter region contains a sequence corresponding to the AR/PR binding site.

To test whether the expression of these miRNAs is influenced by the studied insecticides, we treated MCF-7 cells with *o,p′*-DDT, *p,p′*-DDT or endosulfan. We also treated MDA-MB-231 cells to investigate whether the observed changes in miRNA expression were present in ER-, PR-, AR-negative cells.

**Figure 1:**
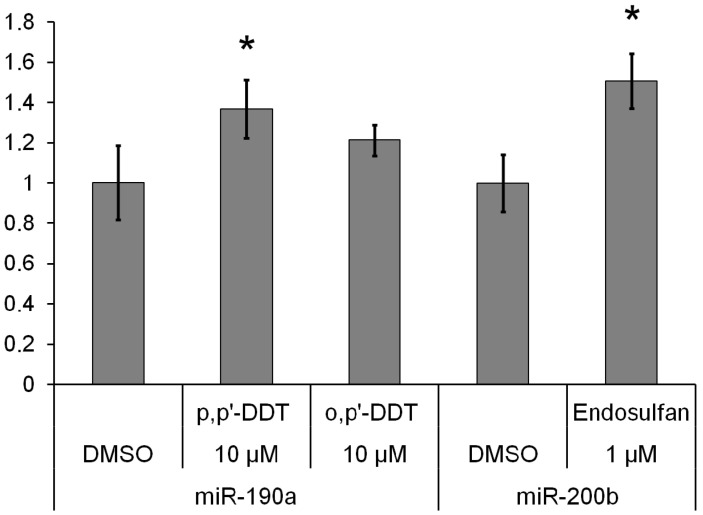
Changes in relative levels of miRNAs in MDA-MB-231 cells exposed to an insecticide for 48 h. The *Y*-axis shows the ratio of the miRNA level in the cells treated with a DDT isomer or endosulfan to the miRNA level in the cells treated with DMSO. Each value is the mean of three independent experiments. * Significant differences from the control (*p* < 0.05).

**Figure 2:**
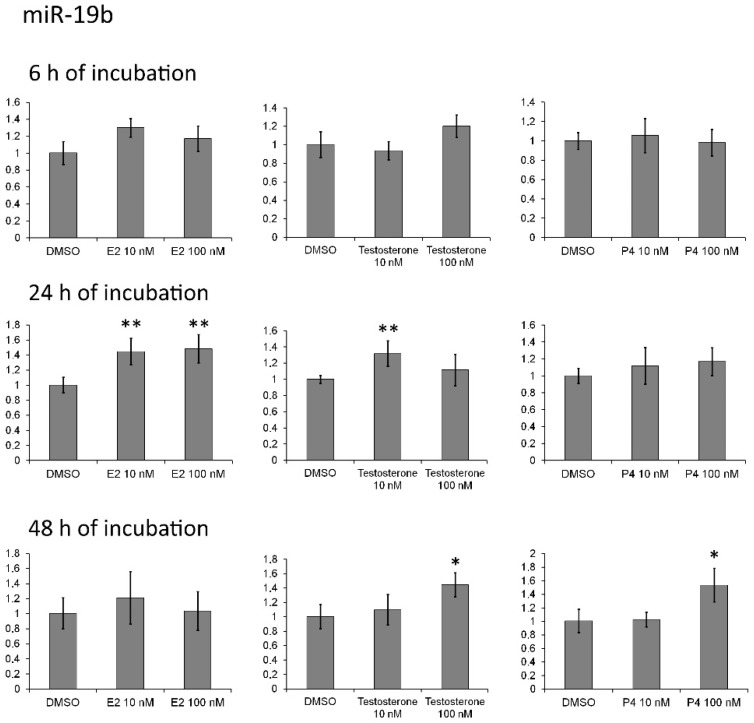
Changes in relative level of miR-19b in MCF-7 cells exposed to estradiol (E2), progesterone (P4) or testosterone. The *Y*-axis shows the ratio of the miRNA level in the cells treated with a hormone to the miRNA level in the cells treated with DMSO. Each value is the mean of three independent experiments. * Significant differences from the control (*p* < 0.05); ** *p* < 0.01.

Incubation of MCF-7 cells with 10 μM *p,p′*-DDT caused a 1.4-fold increase in the miR-27a level (Table 7). Treatment of the cells with 10 μM *o,p′*-DDT for 24 or 48 h resulted in 1.3-fold upregulation of estradiol-sensitive miR-190b. The relative level of miR-190a declined 1.4–1.6-fold in the cells that were incubated with 0.1 or 10 μM *o,p′*-DDT for 48 h. A small increase in the miR-190a level after 48 h of incubation with the high dose of *p,p′*-DDT was observed in both MCF-7 and MDA-MB-231 cells (Figure 1). The amount of miR-200b also went up under the influence of endosulfan in both cell lines. The levels of miR-126 and miR-378a were not affected by the hormones but increased 1.3–1.4-fold in cells that were incubated for 6 h with 10 μM *o,p′*-DDT or *p,p′*-DDT. The expression of miR-193b rose after incubation with 10 μM *p,p′*-DDT for 6 h (1.3-fold) and after treatment with 10 μM *o,p′*-DDT for 48 h (1.4-fold). The level of progesterone- and estradiol-responsive miR-324 increased 1.3–1.4-fold in the cells after incubation with *o,p′*-DDT for 6 h. The expression of miR-342 went up after 24 and 48 h treatment with 10 μM *o,p′*-DDT. The miR-19b amount was elevated in the cells by each hormone (Figure 2) and by the *p,p′*-DDT and endosulfan.

The levels of miR-23a, miR-27a, miR-190b, miR-21, miR-126, miR-378, miR-423, miR-149, miR-193b, miR-324, miR-342, and miR-19b did not change in MDA-MB-231 cells treated with insecticides.

### 3.3. Treatment of Cells with either DDT or Endosulfan Alters Levels of mRNAs That Are Targets of the Studied MiRNAs

We chose genes that are targets of several of the analyzed miRNAs (Table 8). In addition, we were interested in the *PTPRS* (protein tyrosine phosphatase receptor type S), which, according to the TargetScan database, is a target for only 12 miRNAs conserved among vertebrates, including miR-190 [37]. According to The Human Protein Atlas, OXTR (oxytocin receptor) expression is the highest in breasts; expression levels of *AKR1C2* (aldo-keto reductase family 1 member C2), *TRPS1* (transcriptional repressor GATA-binding 1), and *XIAP* (X-linked inhibitor of apoptosis) are moderate or high in breast cells. According to the database information on the expression of *PTPRS* mRNA, breast cells are also characterized by its high expression. All data are available at v21.proteinatlas.org [38].

To determine whether the observed changes in the expression of target genes were the result of hormone-like action of insecticides, we also assessed the mRNA levels in the cells exposed to estradiol, progesterone, or testosterone.

*OXTR* expression decreased after 6 h of incubation with 10 μM *p,p′*-DDT, 10 μM o,p′-DDT, or 10 or 100 nM progesterone (1.6-, 1.8-, and 1.4-fold, respectively; Table 9). Furthermore, the mRNA level of this receptor diminished in the cells treated with *p,p′*-DDT or endosulfan for 48 h. Under the action of estradiol or testosterone, *OXTR* expression increased. *AKR1C2* expression decreased in cells treated with testosterone for 24 and 48 h but rose in cells incubated with 10 μM *p,p′*-DDT. The *TRPS1* mRNA level increased in testosterone- and estradiol-treated cells but declined 1.6-fold in cells treated with the high dose of *o,p′*-DDT for 6 h. *TP53INP1* (tumor protein P53-inducible nuclear protein 1) mRNA expression decreased in cells after 6 h of treatment with either estradiol or *o,p′*-DDT (by 1.4–1.6- and 1.6-fold, respectively). Additionally, the mRNA level of this gene increased under the influence of progesterone after 24 h of incubation and went down 1.5–1.6-fold after 48 h of incubation with *p,p′*-DDT. *APAF1* (apoptotic peptidase-activating factor 1) expression went up in testosterone-treated cells and diminished in cells treated for 6 h with 100 nM progesterone. When cells were exposed to each insecticide, a 1.4-fold decrease in *APAF1* expression was observed after incubation with *p,p′*-DDT for 48 h and after incubation with *o,p′*-DDT for 6 h. The level of *PTPRS* mRNA declined under the influence of testosterone. In cells treated with estradiol, this gene’s expression increased after 24 h of incubation but decreased after 48 h of incubation. During the exposure to each insecticide, *PTPRS* was found to be upregulated after 6 h.

### 3.4. Relative Amounts of Proteins OXTR, APAF1, TP53INP1, and PTPRS in Treated Cells

To confirm that the identified alterations in the expression of target mRNAs were accompanied by changes in the amounts of respective proteins, we performed Western blot analysis of cells exposed to each insecticide for 24 or 48 h. For this assay, we chose OXTR, APAF1, TP53INP1, and PTPRS because their mRNA levels were significantly influenced by several compounds being tested.

**Figure 3:**
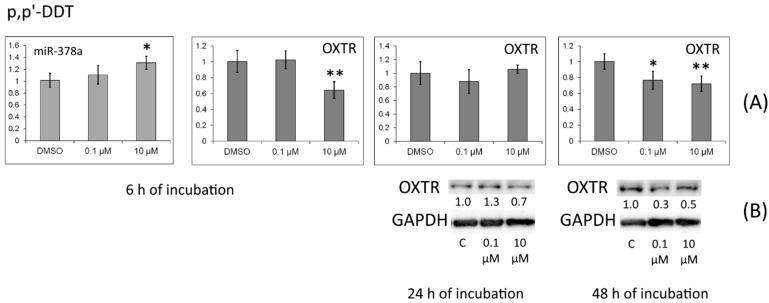
(**A**) Relative levels of *OXTR* mRNA and of the miRNA targeting it (miR-378a) in MCF-7 cells exposed to *p,p′*-DDT. The Y-axis represents the ratio of the mRNA or miRNA amount in the cells treated with a DDT isomer to the mRNA or miRNA amount in the cells treated with DMSO. Each value is the mean of three independent experiments. * Significant differences from the control (*p* < 0.05); ** *p* < 0.01. (**B**) Western blot analysis of OXTR in cells incubated with *p,p′*-DDT. The numbers under OXTR bands denote fold change in the protein level as compared to the first band (control); each data point was normalized to the corresponding GAPDH band. We present data from one of three independent experiments (which yielded similar results).

We confirmed that the amount of the OXTR protein was lower in cells treated with *p,p′*-DDT for 24 and 48 h (Figure 3).

**Figure 4:**
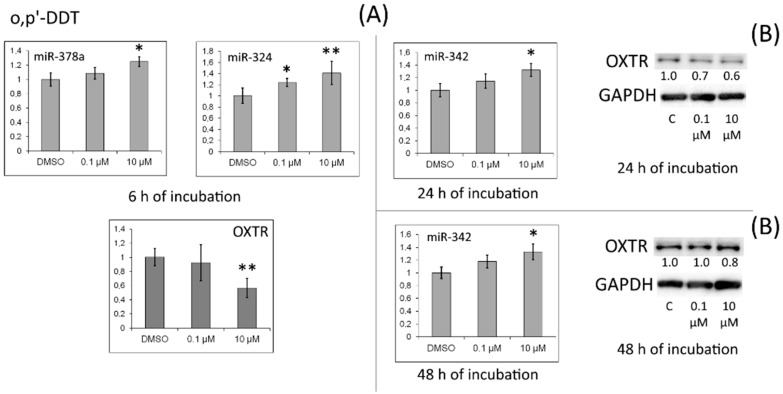
(**A**) Relative levels of *OXTR* mRNA and of miRNAs targeting it (miR-378a, miR-324, and miR-342) in MCF-7 cells exposed to *o,p′*-DDT. The Y-axis shows the ratio of the mRNA or miRNA amount in the cells treated with a DDT isomer to the mRNA or miRNA amount in the cells treated with DMSO. Each value is the mean of three independent experiments. * Significant differences from the control (*p* < 0.05); ** *p* < 0.01. (**B**) Western blot analysis of OXTR in cells incubated with *o,p′*-DDT. The numbers under OXTR bands represent fold change in the protein level as compared to the first band (control); each data point was normalized to the corresponding GAPDH band. We present findings from one of three independent experiments (which yielded similar results).

In cells treated with *o,p′*-DDT for 24 h, the amount of the OXTR protein diminished too, accompanied by upregulation of miR-378a, miR-324, and miR-342 during the exposure to the insecticide (Figure 4).

**Figure 5:**
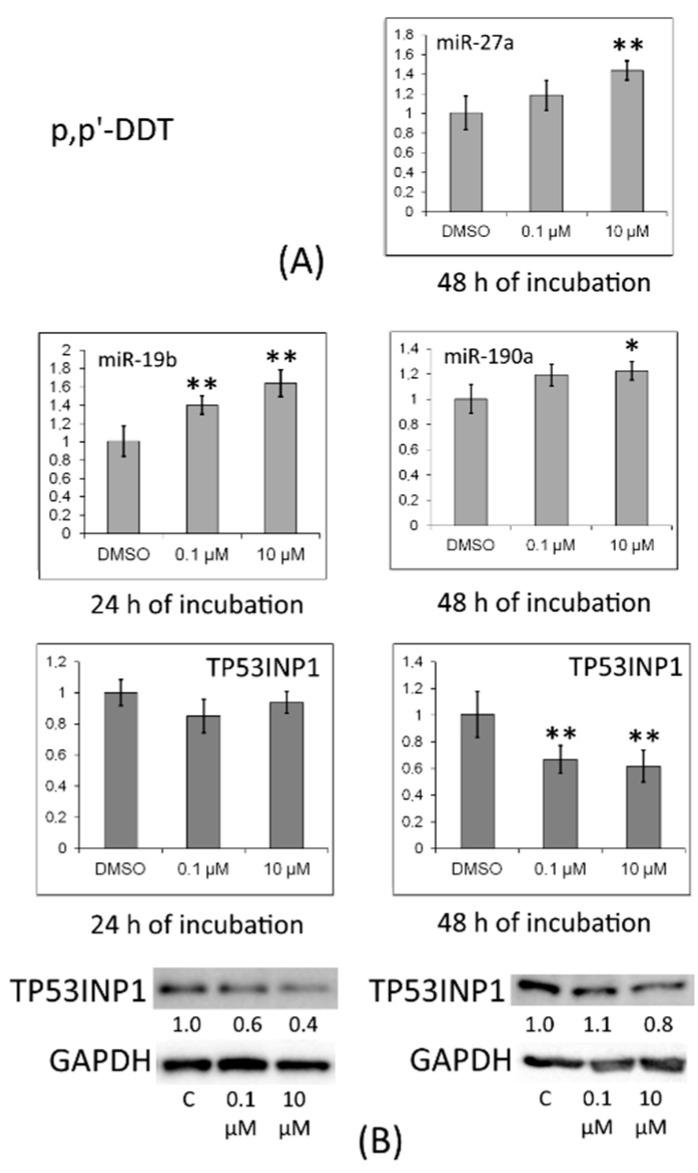
(**A**) Relative levels of *TP53INP1* mRNA and of miRNAs targeting it (miR-19b, miR-27a, and miR-190a) in MCF-7 cells exposed to *p,p′*-DDT. The Y-axis indicates the ratio of the mRNA or miRNA amount in the cells treated with a DDT isomer to the mRNA or miRNA amount in the cells treated with DMSO. Each value is the mean of three independent experiments. * Significant differences from the control (*p* < 0.05); ** *p* < 0.01. (**B**) Western blot analysis of TP53INP1 in cells incubated with *p,p′*-DDT. The numbers under TP53INP1 bands represent fold change in the protein level as compared to the first band (control); each data point was normalized to the corresponding GAPDH band. We present data from one of three independent experiments (which yielded similar results).

The TP53INP1 protein level turned out to be reduced in cells treated with *p,p′*-DDT (Figure 5).

**Figure 6:**
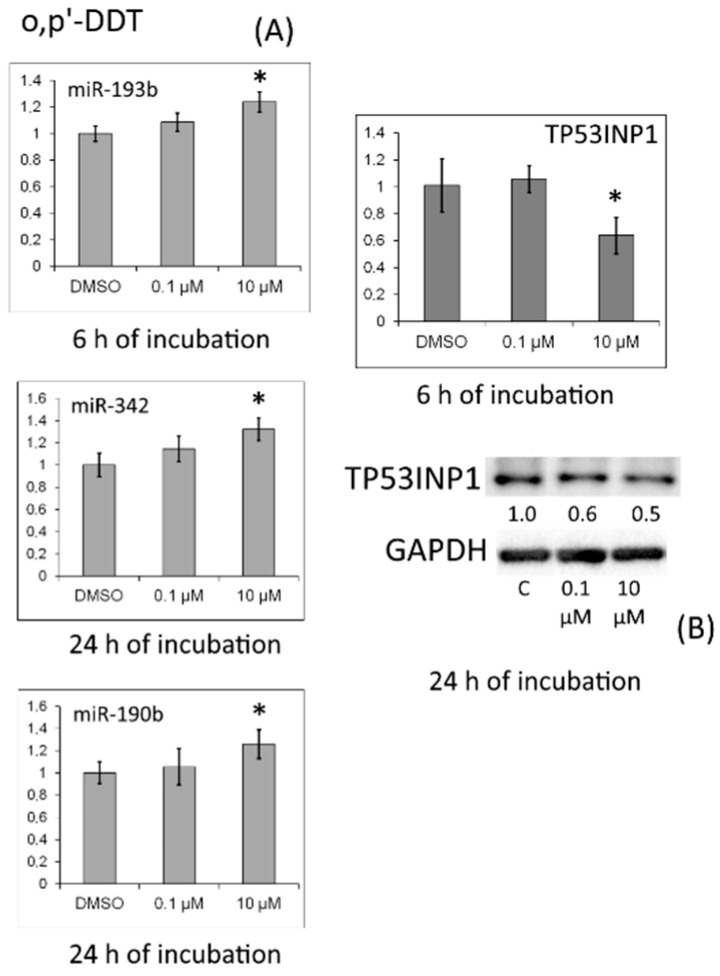
(**A**) Relative levels of *TP53INP1* mRNA and of miRNAs targeting it (miR-193b and miR-342) in MCF-7 cells exposed to *o,p′*-DDT. The Y-axis shows the ratio of the mRNA or miRNA amount in the cells treated with a DDT isomer to the mRNA or miRNA amount in the cells treated with DMSO. Each value is the mean of three independent experiments. * Significant differences from the control (*p* < 0.05). (**B**) Western blot analysis of TP53INP1 in cells incubated with *o,p′*-DDT. The numbers under TP53INP1 bands denote fold change in the protein level as compared to the first band (control); each data point was normalized to the corresponding GAPDH band. We present findings from one of three independent experiments (which yielded similar results).

In cells treated with *o,p′*-DDT for 24 h, this protein’s level declined too (Figure 6).

**Figure 7:**
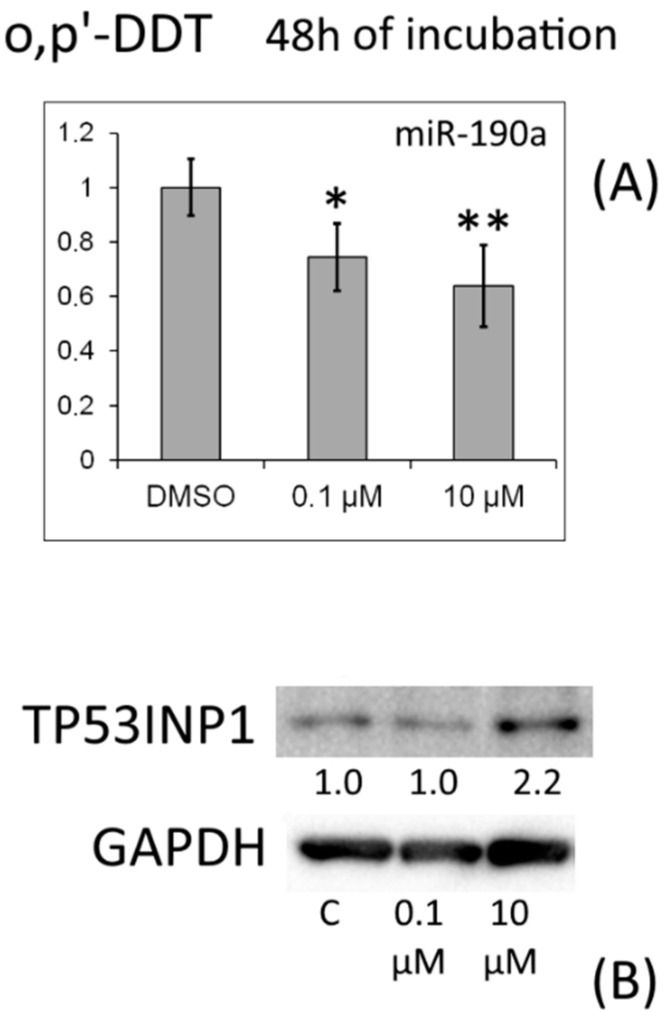
(**A**) Relative level of miR-190a in MCF-7 cells exposed to *o,p′*-DDT for 48 h. The Y-axis shows the ratio of the miRNA amount in the cells treated with a DDT isomer to the miRNA amount in the cells treated with DMSO. Each value is the mean of three independent experiments. * Significant differences from the control (*p* < 0.05); ** *p* < 0.01. (**B**) Western blot analysis of TP53INP1 in cells incubated with *o,p′*-DDT. The numbers under TP53INP1 bands denote fold change in the protein level as compared to the first band (control); each data point was normalized to the corresponding GAPDH band. We present findings from one of three independent experiments (which yielded similar results).

After 48 h of incubation with *o,p′*-DDT, we observed an increase in the amount of TP53INP1 protein, which corresponded to a decrease in the expression of miR-190a (Figure 7).

**Figure 8:**
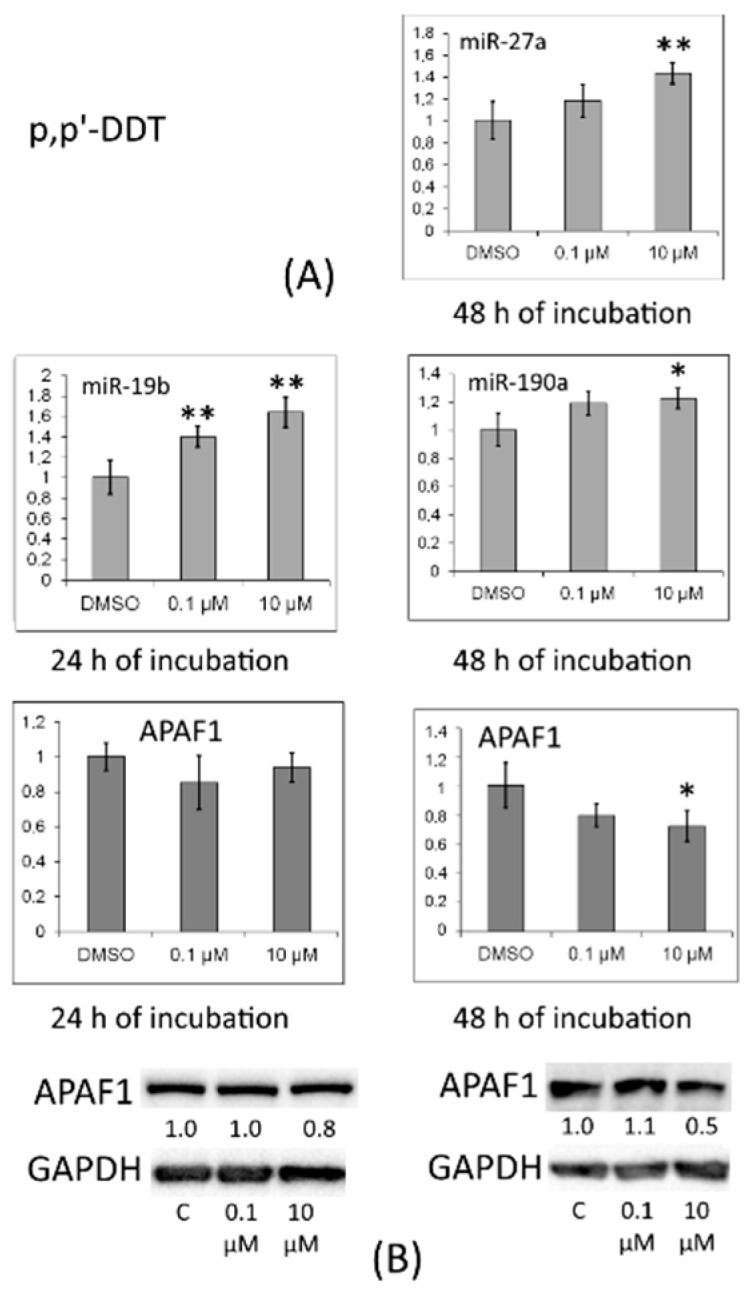
(**A**) Relative levels of *APAF1* mRNA and of miRNAs targeting it (miR-193b and miR-342) in MCF-7 cells exposed to *p,p′*-DDT. The Y-axis represents the ratio of the mRNA or miRNA amount in the cells treated with a DDT isomer to the mRNA or miRNA amount in the cells treated with DMSO. Each value is the mean of three independent experiments. * Significant differences from the control (*p* < 0.05); ** *p* < 0.01. (**B**) Western blot analysis of APAF1 in cells incubated with *p,p′*-DDT. The numbers under APAF1 bands denote fold change in the protein level as compared to the first band (control); each data point was normalized to the corresponding GAPDH band. We present data from one of three independent experiments (which yielded similar results).

The amount of APAF1 diminished under the influence of 10 μM *p,p′*-DDT after 48 h of incubation, consistently with the results of PCR (Figure 8).

**Figure 9:**
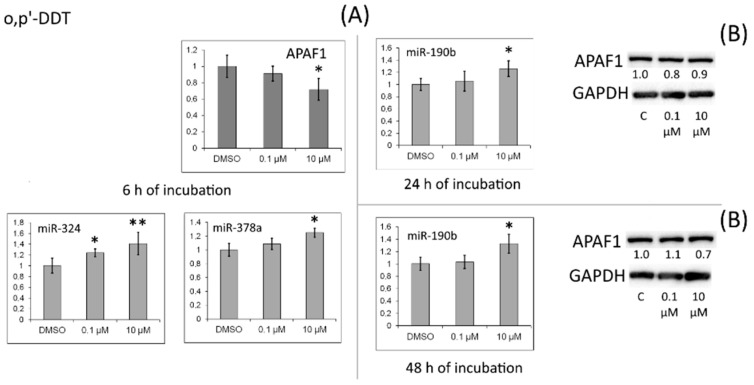
(**A**) Relative levels of *APAF1* mRNA and of miRNAs targeting it (miR-324, miR-378a, and miR-190b) in MCF-7 cells exposed to *o,p′*-DDT. The Y-axis represents the ratio of the mRNA or miRNA amount in the cells treated with a DDT isomer to the mRNA or miRNA amount in the cells treated with DMSO. Each value is the mean of three independent experiments. * Significant differences from the control (*p* < 0.05); ** *p* < 0.01. (**B**) Western blot analysis of APAF1 in cells incubated with *o,p′*-DDT. The numbers under APAF1 bands denote fold change in the protein level as compared to the first band (control); each data point was normalized to the corresponding GAPDH band. We present data from one of three independent experiments (which yielded similar results).

A small decrease in the APAF1 amount occurred in cells after incubation with *o,p′*-DDT (Figure 9).

**Figure 10:**
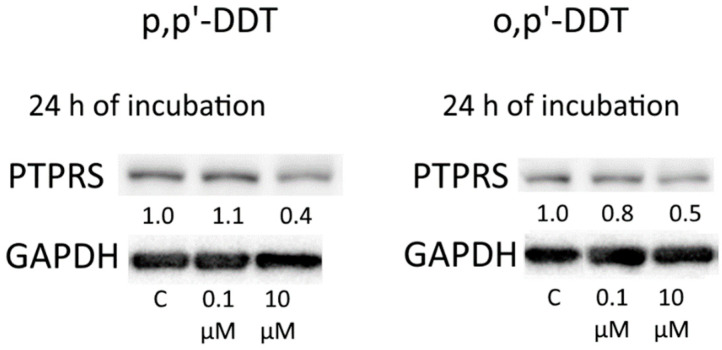
Western blot analysis of PTPRS in cells incubated with *p,p′*-DDT and *o,p′*-DDT. The numbers under PTPRS bands denote fold change in the protein level as compared to the first band (control); each data point was normalized to the corresponding GAPDH band. We present data from one of three independent experiments (which yielded similar results). The levels of proteins APAF1 and OXTR declined in the cells treated with endosulfan for 24 h, and TP53INP1, APAF1, and OXTR levels decreased in the cells treated for 48 h (Figure 11).

Although the *PTPRS* mRNA level proved to be elevated in the cells incubated with each insecticide, there was a decrease in the protein level of PTPRS in the cells incubated with *p,p′*-DDT or *o,p′*-DDT within 24 h (Figure 10).

### 3.5. The Effects of p,p′-DDT, o,p′-DDT, and Endosulfan on Apoptosis of MCF-7 Cells

Since the level of apoptosis regulating proteins decreased under the action of insecticides, we investigated the effect of the compounds on cell apoptosis. The rate of apoptosis was detected by Hoechst 33342/propidium iodide. We did not find significant changes in the rate of apoptosis in cells treated with insecticides. However, it should be noted that a tendency towards a decrease in the rate of apoptosis was observed in cells treated with 1 μM and 10 μM *o,p′*-DDT for 48 h (Table 10). A similar trend was observed in cells treated with 0.1 μM endosulfan.

## 4. Discussion

It is reported that there are ~1000 chemicals that can interfere with the endocrine system [3]. There are difficulties with the research on EDCs’ mechanisms of action and with assessing the consequences of their impact on humans because adverse effects manifest themselves years after EDC exposure and are absent in some people [3]. One of the most famous EDCs is DDT. A number of studies suggest that the exposure to DDT elevates the risk of breast cancer [8,17,41]. Another insecticide with similar properties is endosulfan; it was actively used in agriculture after the prohibition of DDT. How DDT and endosulfan may contribute to cancer has not been well investigated. Elsewhere, we have discovered some estradiol-, progesterone-, and testosterone-sensitive miRNAs [26]. Because DDT and endosulfan have estrogenic, antiandrogenic, and antiprogesterone effects, we hypothesized that these miRNAs can be affected by insecticides. Therefore, we measured relative levels of these miRNAs and expression of their target genes in MCF-7 cells treated with *p,p′*-DDT, *o,p′*-DDT, or endosulfan. Furthermore, we analyzed the expression of miR-19b, which is known to be regulated by estrogen [36,42], and miRNA genes in whose promoter regions sequences matching ER, PR, or AR binding sites have been found but expression levels were not altered by hormones. Here, we also revealed that the promoter region of *MIR19b* contains a binding site for AR and PR.

We noticed that the treatment of cells with *p,p′*-DDT alters the expression of miR-27a, miR-190a, miR-126, miR-378a, miR-193b, and miR-19b. MiR-190a was also upregulated in ER-, PR-, AR-negative MDA-MB-231 cells by this insecticide, probably indicating ER-, PR-, AR-independent effects of *p,p′*-DDT on this miRNA’s expression. It is noteworthy that *p,p′*-DDT and testosterone caused opposite alterations of miR-27a expression. Similarly, the level of miR-190a went down in cells after incubation with progesterone but increased after the exposure to *p,p′*-DDT, which has the strongest affinity for PR as compared to other DDT isomers and endosulfan (potency of *p,p′*-DDT binding to PR is only 40-fold weaker than that of progesterone) [10]. Amounts of estradiol-responsive miR-19b and miR-193b went up in the cells exposed to *p,p′*-DDT.

Treatment of cells with *o,p′*-DDT resulted in overexpression of estradiol-sensitive miR-190b and miR-193b and progesterone- and estradiol-responsive miR-324. The levels of miR-126, miR-378a, miR-423, and miR-342 rose too. MiR-190a expression, which increased under the action of testosterone, significantly decreased in cells treated with *o,p′*-DDT.

MiR-200b was upregulated by the exposure to endosulfan in both MCF-7 and MDA-MB-231 cells. A small increase was observed for miR-19b level in cells treated with endosulfan for 6 h.

For further analysis, we selected miRNA target genes *OXTR*, *AKR1C2*, *TRPS1*, *TP53INP1*, *APAF1*, *XIAP*, and *PTPRS*. The revealed increase in the *AKR1C2* mRNA level under the influence of *p,p′*-DDT is probably related to antiandrogenic properties of DDT because we found that testosterone treatment of the cells lowers this gene’s mRNA level. The AKR1C2 protein is involved in the metabolism of hormones such as estrogen, progesterone, and androgen. Its expression change caused by DDT is probably another way that DDT affects the endocrine system.

*OXTR* mRNA levels were reduced in the cells by the exposure to progesterone, *p,p′*-DDT, or *o,p′*-DDT for 6 h. On the other hand, incubation with progesterone or *o,p′*-DDT raised the expression of miR-324, and treatment with *p,p′*-DDT or *o,p′*-DDT upregulated miR-378. *OXTR* mRNA is one of the targets of these miRNAs. We confirmed that under the action of *p,p′*-DDT or *o,p′*-DDT, the protein level of *OXTR* decreased.

MRNA levels of proapoptotic antiproliferative protein TP53INP1 were reduced in the cells by treatment with *p,p′*-DDT for 48 h or *o,p′*-DDT for 6 h. The observed alterations may be due to estrogenic and antiprogesterone properties of DDT. Nonetheless, it should be noted that after 24 h of incubation with *p,p′*-DDT, the level of miR-19b increased, and after incubation for 48 h, the levels of miR-27a and miR-190a went up. For all these miRNAs, *TP53INP1* mRNA is predicted as a target. In cells treated with *o,p′*-DDT for 6 h, the miR-193b amount increased, and levels of miR-342 and miR-190b increased after 24 h of incubation; for these miRNAs, *TP53INP1* mRNA is also predicted to be a target. We confirmed by Western blotting the TP53INP1 protein downregulation under the influence of *p,p′*-DDT and *o,p′*-DDT (after 24 h of incubation). After 48 h of incubation with *o,p′*-DDT, the amount of TP53INP1 protein increased, which corresponded to a decrease in miR-190a expression.

The mRNA expression of *APAF1*, the protein product of which participates in apoptosis initiation, was significantly reduced in cells by incubation with *p,p′*-DDT (for 48 h) or *o,p′*-DDT (for 6 h). This mRNA, just as *TP53INP1* mRNA, is a target for miR-19b, miR-190a and miR-27a.

It was shown previously that incubation with *o,p′*-DDT for 5 days reduces the rate of apoptosis [43]. Other researchers have demonstrated that *o,p′*-DDT has the ability to suppress tumor necrosis factor alpha (TNF)-induced apoptosis in MCF-7 cells [44]. Thus, the previously demonstrated inhibition of apoptosis by this insecticide may be mediated, among other things, by a decrease in the expression of APAF1 and TP53INP1.

Although we failed to detect significant changes in the levels of miRNAs or their target mRNAs (with the exception of *OXTR*, miR-200b, and miR-19b) in cells exposed to endosulfan, we demonstrated that after incubation with this insecticide, protein levels of TP53INP1, APAF1, and OXTR decreased.

## 5. Conclusions

Thus, we identified new targets of action of the insecticides *p,p′*-DDT, *o,p′*-DDT, and endosulfan. Treatment of MCF-7 breast cancer cells with these compounds lowered the expression of regulators of apoptosis TP53INP1 and APAF1 (but after 48 h of incubation with *o,p′*-DDT, the TP53INP1 amount increased). The expression of *OXTR*, which is probably implicated in breast carcinogenesis [45,46], significantly changed as well. In the cells treated with *p,p′*-DDT or *o,p′*-DDT, the observed alterations were accompanied by quantitative changes in the hormone-responsive miRNAs targeting these mRNAs (miR-324, miR-190a, miR-190b, miR-27a, miR-193b, and miR-19b).

## Figures and Tables

**Figure 11:**
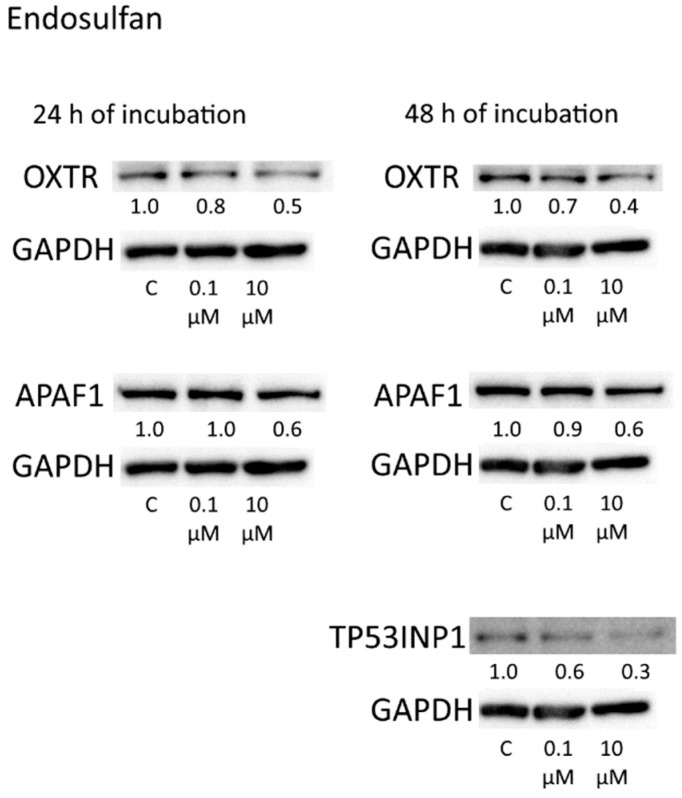
Western blot analysis of OXTR, APAF1, and TP53INP1 in cells incubated with endosulfan for 24 and 48 h. The numbers under bands denote fold change in the protein level as compared to the first band (control); each data point was normalized to the corresponding GAPDH band. We present data from one of three independent experiments (which yielded similar results).

**Table 1:** Sequences of primers for miRNA reverse transcription.

RNA	Primer Sequences
hsa-miR-23a-3p	5′-GTCGTATCCAGTGCAGGGTCCGAGGTATTCGCACTGGATACGACGGAAATC-3′
hsa-miR-190a-5p	5′-GTCGTATCCAGTGCAGGGTCCGAGGTATTCGCACTGGATACGACACCTAATA-3′
hsa-miR-190b-5p	5′-GTCGTATCCAGTGCAGGGTCCGAGGTATTCGCACTGGATACGACAACCCAA-3′
hsa-miR-27a-3p	5′-GTCGTATCCAGTGCAGGGTCCGAGGTATTCGCACTGGATACGACTGCTCACA-3′
hsa-miR-193b-3p	5′-GTCGTATCCAGTGCAGGGTCCGAGGTATTCGCACTGGATACGACAGCGGGAC-3′
hsa-miR-324-5p	5′-GTCGTATCCAGTGCAGGGTCCGAGGTATTCGCACTGGATACGACACACCAAT-3′
hsa-miR-423-3p	5′-GTCGTATCCAGTGCAGGGTCCGAGGTATTCGCACTGGATACGACACTGAGGG-3′
hsa-miR-200b-3p	5′-GTCGTATCCAGTGCAGGGTCCGAGGTATTCGCACTGGATACGACGTCATCAT-3′
hsa-miR-21-5p	5′-GTCGTATCCAGTGCAGGGTCCGAGGTATTCGCACTGGATACGACTCAACATC-3′
hsa-miR-126-3p	5′-GTCGTATCCAGTGCAGGGTCCGAGGTATTCGCACTGGATACGACCGCATTAT-3′
hsa-miR-378a-3p	5′-GTCGTATCCAGTGCAGGGTCCGAGGTATTCGCACTGGATACGACGCCTTCT-3′
hsa-miR-149-5p	5′-GTCGTATCCAGTGCAGGGTCCGAGGTATTCGCACTGGATACGACGGGAGTGA-3′
hsa-miR-342-3p	5′-GTCGTATCCAGTGCAGGGTCCGAGGTATTCGCACTGGATACGACACGGGTG-3′
hsa-miR-19b-3p	5′-GTCGTATCCAGTGCAGGGTCCGAGGTATTCGCACTGGATACGACTCAGTTT-3′
U44	5′-GTCGTATCCAGTGCAGGGTCCGAGGTATTCGCACTGGATACGACAGTCAGTT-3′
U48	5′-GTTGGCTCTGGTGCAGGGTCCGAGGTATTCGCACCAGAGCCAACGGTCAG-3′

**Table 2:** Sequence of primers for RT-PCR of miRNAs.

RNA		Primer Sequences
hsa-miR-23a-3p	Forward	5′-GCCGCATCACATTGCCAGG-3′
	Probe	5′-(R6G)-TTCGCACTGGATACGACGGAAATC-(BHQ1)-3′
hsa-miR-190a-5p	Forward	5′-GCCGCTGATATGTTTGATA-3′
	Probe	5′-(R6G)-TTCGCACTGGATACGACACCTAATA-(BHQ1)-3′
hsa-miR-190b-5p	Forward	5′-GCCGCTGATATGTTTGATA-3′
	Probe	5′-(R6G)-TTCGCACTGGATACGACAACCCAA-(BHQ1)-3′
hsa-miR-27a-3p	Forward	5′-GCCGCTTCACAGTGGCTAA-3′
	Probe	5′-(R6G)-TTCGCACTGGATACGACGCGGAAC-(BHQ1)-3′
hsa-miR-193b-3p	Forward	5′-GCCGCAACTGGCCCTCAAA-3′
	Probe	5′-(R6G)-TTCGCACTGGATACGACAGCGGGAC-(BHQ1)-3′
hsa-miR-324-5p	Forward	5′-CCCGCATCCCCTAGGGC-3′
	Probe	5′-(R6G)-TTCGCACTGGATACGACACACCAAT-(BHQ1)-3′
hsa-miR-423-3p	Forward	5′-GCCGAGCTCGGTCTGAGGC-3′
	Probe	5′-(R6G)-TTCGCACTGGATACGACACTGAGG-(BHQ1)-3′
hsa-miR-200b-3p	Forward	5′-GCCGCTAATACTGCCTGGTA-3′,
	Probe	5′-(R6G)-TTCGCACTGGATACGACGTCATCAT-(BHQ1)-3′
hsa-miR-21-5p	Forward	5′-GCCGCTAGCTTATCAGACT-3′
	Probe	5′-(R6G)-TTCGCACTGGATACGACTCAACATC-(BHQ1)-3′
hsa-miR-126-3p	Forward	5′-GCCGCTCGTACCGTGAGTA-3′
	Probe	5′-(R6G)-TTCGCACTGGATACGACCGCATTAT-(BHQ1)-3′
hsa-miR-378a-3p	Forward	5′-GCCGCACTGGACTTGGAGTC-3′
	Probe	5′-(R6G)-TTCGCACTGGATACGACGCCTTCT-(BHQ1)-3′
hsa-miR-149-5p	Forward	5′-GCCGTCTGGCTCCGTGTCT-3′
	Probe	5′-(R6G)-TTCGCACTGGATACGACGGGAGTGA-(BHQ1)-3′
hsa-miR-342-3p	Forward	5′-GCCGCTCTCACACAGAAATCG-3′
	Probe	5′-(R6G)-TTCGCACTGGATACGACACGGGTGC-(BHQ1)-3′
hsa-miR-19b-3p	Forward	5′-GCCGTGTGCAAATCCATGCA-3′
	Probe	5′-(R6G)-TTCGCACTGGATACGACTCAGTTT-(BHQ1)-3′
U44	Forward	5′-GCCGCTCTTAATTAGCTCT-3′
	Probe	5′-(R6G)-TTCGCACTGGATACGACAGTCAGTT-(BHQ1)-3′
U48	Forward	5′-GAGTGATGATGACCCCAGGTAA-3′
	Probe	5′-(R6G)-TTCGCACCAGAGCCAACGGTCAG-(BHQ1)-3′
	Reverse	5′-GTGCAGGGTCCGAGGT-3′

**Table 3:** Sequence of primers for gene expression analysis by RT-PCR.

Gene		Primer Sequences
*GAPDH*	Forward	5′-ACAACTTTGGTATCGTGGAAGGAC-3′
	Reverse	5′-CAGGGATGATGTTCTGGAGAGC-3′
*ANKRD17*	Forward	5′-AATGTTGCCACCACTCTTCC-3′
	Reverse	5′-TGCAGCTGTGCATTCTTTTC-3′
*SYMPK*	Forward	5′-GCTGGAGAAGAAAGAGGTG-3′
	Reverse	5′-ACAGGTTGGTGGCTTTGATG-3′
*AKR1C2*	Forward	5′-CCTAAAAGTAAAGCTCTAGAGGCCGT-3′
	Reverse	5′-GAAAATGAATAAGATAGAGGTCAACATAG-3′
*TRPS1*	Forward	5′-GTATCCTGCATCGGGAGAAA-3′
	Reverse	5′-AGCTTCTGGTAGAGGCCACA-3′
*TP53INP1*	Forward	5′-GCACCCTTCAGTCTTTTCCTGTT-3′
	Reverse	5′-GGAGAAAGCAGGAATCACTTGTATC-3′
*APAF1*	Forward	5′-AACCAGGATGGGTCACCATA-3′
	Reverse	5′-ACTGAAACCCAATGCACTCC-3′
*XIAP*	Forward	5′-TGGCAGATTATGAAGCACGGATC-3′
	Reverse	5′-AGTTAGCCCTCCTCCACAGTGA-3′
*OXTR*	Forward	5′-GCACGGTCAAGATGACTTTC-3′
	Reverse	5′-GCATGTAGATCCAGGGGTTG-3′

**Table 4:** The cytotoxicity (LC_50_) of *p*,*p′*-DDT, *o*,*p′*-DDT, and endosulfan against MCF-7 cells.

Time, h	LC_50,_ μM		
	*p*,*p′*-DDT	*o*,*p′*-DDT	Endosulfan
6	>50	>50	>50
24	>50	>50	33.6 ± 0.8
48	48.7 ± 0.5	>50	32.8 ± 0.9

**Table 5:** The proliferation (IC_50_) of *p*,*p′*-DDT, *o*,*p′*-DDT, and endosulfan against MCF-7 cells.

Time, h	IC_50,_ μM		
	*p*,*p′*-DDT	*o*,*p′*-DDT	Endosulfan
6	>50	>50	>50
24	68.7 ± 0.8	>50	60.1 ± 0.6
48	65.7 ± 0.7	46.9 ± 0.5	20.7 ± 0.4

**Table 6:** The miRNAs potentially regulated by ER, PR, and AR. Data from [26].

miRNA	Estradiol	Testosterone	Progesterone	Potentially Regulated by
miR-23a	-	-	-	ER, AR
miR-27a	-	↓	-	ER, AR
miR-190b	↓ (6 h)	-	↑	ER, AR, PR
	↑ (24 h)			
miR-190a		↑	↓	ER, PR, AR
miR-200b	↓	↑	-	ER, AR
miR-21	-	↓ (6 h)	↑	ER, PR, AR
		↑ (48 h)		
miR-126	-	-	-	ER, PR, AR
miR-378	-	-	-	ER, PR, AR
miR-423		↑		ER, PR, AR
miR-149	-	-	-	ER, PR, AR
miR-193b	↑	↑	-	ER, PR, AR
miR-324	↑	↑	↑	ER, PR, AR
miR-342	-	-	-	ER, PR, AR

MCF-7 cells were treated with 10 nM or 100 nM of hormone for 6, 24, and 48 h. “↑” and “↓” indicate how miRNA expression changed under the action of a compound (increased or decreased). The incubation time with the compound is indicated in parentheses.

**Table 7:** Relative miRNA levels in MCF-7 cells treated with *p,p′*-DDT, *o,p′*-DDT, or endosulfan.

miRNA	Time, h	Relative level of miRNA							
		*p,p′*-DDT			*o,p′*-DDT			Endosulfan	
		0.1 μM	10 μM		0.1 μM	10 μM		0.1 μM	1 μM
miR-23a	6	0.85	0.92		0.87	1.01		0.83	0.90
	24	0.99	0.98		1.10	1.03		0.91	0.93
	48	1.08	1.20		0.86	0.99		0.96	1.03
miR-27a	6	0.85	0.92		1.14	1.20		0.90	0.95
	24	1.08	1.00		0.97	1.01		1.05	1.15
	48	1.19	1.44 **		0.94	1.09		1.08	1.07
miR-190b	6	0.93	0.91		0.96	1.07		0.96	1.06
	24	0.98	1.02		1.05	1.26 *		0.98	1.16
	48	0.87	0.91		1.00	1.33 *		1.08	1.06
miR-190a	6	0.90	0.98		0.91	0.93		1.08	1.09
	24	1.01	0.91		0.95	0.94		0.94	1.01
	48	1.19	1.23 *		0.73 *	0.63 **		0.97	1.07
miR-200b	6	1.06	1.17		1.02	1.02		1.06	0.97
	24	1.08	1.11		1.07	1.01		0.99	0.99
	48	1.02	1.08		1.04	1.15		1.08	1.28 *
miR-21	6	0.97	1.11		1.03	1.07		1.06	1.04
	24	1.10	1.11		1.03	1.03		1.11	1.16
	48	0.91	0.97		1.05	1.10		1.01	1.19
miR-126	6	1.14	1.27 *		1.04	1.38 **		1.03	1.01
	24	1.13	0.92		1.00	0.95		1.01	1.17
	48	0.87	0.90		1.07	1.09		1.13	1.05
miR-378	6	1.11	1.31 *		1.09	1.25 *		1.03	1.10
	24	1.00	1.14		0.95	1.00		0.97	1.11
	48	1.07	1.11		1.05	1.21		1.11	1.04
miR-423	6	1.09	1.17		0.85	1.05		0.94	0.88
	24	1.08	1.08		1.17	1.26 *		0.89	0.92
	48	0.95	1.03		0.94	1.17		0.97	1.00
miR-149	6	0.97	1.16		1.09	1.01		1.08	1.13
	24	1.08	1.14		0.89	1.00		0.94	0.95
	48	1.03	1.05		0.83	0.82		1.13	1.06
miR-193b	6	1.04	1.28 *		1.08	1.24 *		0.99	1.09
	24	1.02	0.97		0.88	0.97		0.95	1.08
	48	1.00	1.03		1.02	1.40 **		1.00	0.91
miR-324	6	0.98	1.03		1.25 *	1.42 **		0.94	0.91
	24	1.09	0.96		1.10	1.04		0.92	1.05
	48	1.00	0.95		0.98	0.95		1.08	1.14
miR-342	6	1.00	0.91		0.89	1.01		1.09	0.94
	24	0.96	1.16		1.15	1.32 *		0.84	0.98
	48	0.90	0.96		1.18	1.33 *		1.16	1.03
miR-19b	6	0.84	1.01		0.96	0.99		1.18	1.23 *
	24	1.40 **	1.64 **		1.03	0.96		1.12	1.11
	48	0.98	1.15		0.99	1.08		0.91	0.82

Each value represents the mean of three independent experiments; the results are normalized to the control (cells treated with dimethyl sulfoxide; DMSO). Significant differences are highlighted in bold. * Significant differences from the control (*p* < 0.05); ** *p* < 0.01.

**Table 8:** The studied miRNAs and their target mRNAs in humans.

Target mRNA	miRNAs
*OXTR*	miR-23a-3p, miR-21-5p, **miR-378a-3p**, miR-149-5p, **miR-324-5p**, **miR-342-3p**
*AKR1C2*	miR-23a-3p, **miR-27a-3p**, **miR-190-5p**, miR-21-5p, **miR-342-3p**, miR-193b-3p *
*TRPS1*	miR-23a-3p, **miR-27a-3p**, **miR-190-5p**, miR-200b-3p, **miR-193-3p**, miR-149-5p, **miR-324-5p**, **miR-19-3p**
*TP53INP1*	miR-23a-3p, **miR-27a-3p**, miR-190-5p **, miR-200b-3p, **miR-193-3p**, **miR-342-3p**, **miR-19-3p**
*APAF1*	miR-23a-3p *, **miR-27a-3p**, **miR-190-5p**, miR-200b-3p, miR-21-5p *, **miR-378a-3p**, miR-149-5p, **miR-324-5p**, **miR-19-3p**
*XIAP*	miR-23a-3p, **miR-27a-3p**, **miR-190-5p**, miR-200b-3p *, miR-149-5p, **miR-19-3p**
*PTPRS*	**miR-190-5p**

The miRNAs with the largest number of detected expression alterations in the cells treated with the insecticides are highlighted in bold. * The interaction of miRNA with the target mRNA was confirmed by a reporter assay according to the miRTarBase database [39]. ** The interaction of miRNA with the target mRNA was confirmed by a reporter assay [40].

**Table 9:** Relative mRNA levels in MCF-7 cells exposed to estradiol, testosterone, progesterone, *p,p′*-DDT, *o,p′*-DDT, or endosulfan.

Gene	Time, h	Relative Level of mRNA											
		Estradiol		Testosterone		Progesterone		*p,p′*-DDT		*o,p′*-DDT		Endosulfan	
		10 nM	100 nM	10 nM	100 nM	10 nM	100 nM	0.1 μM	10 μM	0.1 μM	10 μM	0.1 μM	1 μM
*OXTR*	6	0.81	1.01	0.97	1.02	0.70 *	0.71 *	1.02	0.64 **	0.92	0.57 **	0.96	0.90
	24	1.25 *	1.31 *	1.24	1.73 **	1.00	1.00	0.88	1.06	1.10	1.00	1.05	0.88
	48	1.57 *	2.45 **	**1.25** *	1.26 *	1.16	1.33 *	0.77 *	0.72 **	1.03	0.98	0.86	0.67 **
*AKR1C2*	6	1.03	1.09	1.05	1.12	0.80	0.83	0.98	0.93	0.93	0.81	0.97	0.99
	24	1.00	0.90	0.67 *	0.71 *	0.93	0.94	1.00	1.27 *	0.96	1.18	0.88	1.22
	48	0.93	1.23	0.74 *	0.65 **	0.91	0.90	0.99	1.42 **	0.83	1.10	0.94	1.01
*TRPS1*	6	1.04	1.11	1.16	1.11	0.85	0.97	0.98	0.90	0.98	0.63 **	0.97	1.01
	24	1.19	0.99	1.06	1.09	1.07	1.02	0.90	1.00	1.02	1.17	1.06	0.98
	48	1.12	1.62 **	1.35 *	1.42 **	1.12	1.12	0.88	0.81	1.05	1.03	0.88	1.07
*TP53INP1*	6	0.64 **	0.71 *	1.17	1.12	0.93	0.94	1.08	0.87	1.06	0.64 *	1.00	0.98
	24	0.83	0.85	0.82	0.88	1.31 *	1.29 *	0.85	0.94	1.10	1.02	1.11	1.03
	48	0.80	**0.** 78 *	0.87	1.00	1.05	1.11	0.67 **	0.62 **	1.08	0.96	0.96	0.98
*APAF1*	6	0.95	1.15	1.26 *	1.39 *	0.84	0.73 **	1.14	1.22 *	0.91	0.72 *	0.92	1.00
	24	1.15	1.07	0.95	0.96	1.20	1.18	0.85	0.94	1.07	1.16	1.17	1.06
	48	0.88	0.95	0.78	0.99	1.06	1.07	0.80	0.73 *	1.02	0.87	1.01	1.07
*XIAP*	6	0.81	0.95	0.98	1.04	0.86	1.10	1.10	0.93	1.04	0.83	0.88	0.82
	24	1.01	0.97	0.95	0.91	1.07	1.12	1.03	1.09	1.10	0.96	1.00	0.97
	48	1.17	1.13	1.02	1.11	1.08	1.05	0.93	0.82	0.98	0.77 *	1.06	0.93
*PTPRS*	6	1.14	1.20	1.14	1.01	0.90	0.90	1.11	1.34 *	1.35 *	1.72 **	1.01	1.28 *
	24	1.27 *	1.33 *	0.75 *	0.79 *	1.11	1.12	1.12	1.03	0.99	1.01	1.17	1.06
	48	0.76 *	0.66 **	0.80	0.79 *	1.19	1.21	1.07	1.13	1.15	1.26	0.84	0.92

Each value is the mean of three independent experiments; the results are normalized to the control (cells treated with DMSO). Significant differences are highlighted in bold. * Significant differences from the control (*p* < 0.05); ** *p* < 0.01.

**Table 10:** Percentage of dead, living and apoptotic cells after incubation for 48 h with DMSO, *o,p′*-DDT or endosulfan.

Dose	*o,p′*-DDT			Endosulfan		
	Dead Cells (%)	Live Cells (%)	Apoptosis (%)	Dead Cells (%)	Live Cells (%)	Apoptosis (%)
0 μM	0.19 ± 0.19	96.99 ± 0.08	2.82 ± 0.20	0.19 ± 0.19	96.99 ± 0.08	2.82 ± 0.20
0.1 μM	1.12 ± 0.97	96.61 ± 2.35	2.27 ± 1.38	0.55 ± 0.40	97.87 ± 1.13	1.59 ± 0.83
1 μM	0.22 ± 0.20	97.75 ± 0.07	2.03 ± 0.38	0.54 ± 0.22	96.90 ± 0.56	2.56 ± 0.34
10 μM	0.50 ± 0.24	97.83 ± 1.19	1.67 ± 0.95	0.52 ± 0.31	95.59 ± 1.12	3.89 ± 0.80

## Data Availability

The data presented in this study are available on request from the corresponding author.
